# Nursing Leaders’ Knowledge and Awareness of Bullying and Lateral Violence: A Qualitative Study

**DOI:** 10.1177/23779608241274210

**Published:** 2024-08-16

**Authors:** Corina Elena Luca, Giovanna Pezzoli, Stefan Kunz, Monica Bianchi

**Affiliations:** 1 Regional Hospital of Lugano, Ente Ospedaliero Cantonale, Lugano, Switzerland; 2 Department of Business Economics, Health and Social Care, University of Applied Sciences and Arts of Southern Switzerland, Manno, Switzerland

**Keywords:** bullying, lateral violence, leadership, workforce issues, nursing, qualitative approaches

## Abstract

**Introduction:**

Bullying and lateral violence are prevalent phenomena within the nursing profession, exerting significant impacts on patient safety, the nursing profession and the organisation. The pivotal role of nurse leaders is paramount in both the prevention and resolution of these issues.

**Aim:**

The aim is to explore the level of awareness and knowledge of bullying and lateral violence of nurse leaders in a public hospital in Switzerland.

**Methods:**

A qualitative descriptive study has been conducted. Data were collected from February to August 2020 using semi-structured interviews and focus groups that were recorded and transcribed verbatim. Two researchers independently utilised Braun and Clarke's thematic analysis to code, categorise and synthesise the data. The sample of nursing middle-management leaders was purposive.

**Results:**

The study involved 35 nurse leaders as participants. Through data analysis, 15 themes were identified, which were further grouped into five major themes: characteristics of the phenomena, facilitating and hindering factors, emotions/experiences, strategies and supports. The results highlighted that nurse leaders may have a lack of knowledge about these phenomena, leading to challenges in their identification. The awareness achieved by the nurse leaders highlighted their need to understand what they were ‘fighting against’.

**Conclusion:**

It is essential to consider the impact of these phenomena on employees’ well-being and their potential consequences for patient safety, quality of care and financial performance. A preventive approach by increasing nurse leaders’ competence in observing everyday working realities and identifying strategies for addressing bullying is required. Further research on the construction and implementation of specific interventions is essential, aimed at preventing and addressing these phenomena comprehensively.

## Introduction

Bullying and lateral violence are pervasive issues among nurses (Bambi et al., 2017; Bambi, Guazzini et al., 2019; Crawford et al., 2019; Germann & Moore, 2019; Karatuna et al., 2020). The prevalence of these phenomena within the nursing profession is notably high, with reported rates ranging from 67.5% to 90.4% for incivility, from 1% to 87.4% for lateral violence. In comparison, bullying prevalence varies between 2.4% and 81% (Bambi et al., 2018). Moreover, variations in lateral violence prevalence across different regions suggest possible influences from cultural environments (Zhang et al., 2022). The American Nurses Association (ANA) has classified these behaviours as an ‘epidemic’ and has issued a ‘sentinel alert’ against any conduct that undermines the culture of safety (Kroning, 2019). The Nurses’ Code of Ethics also highlights the importance of developing strategies and guidelines to address work-related issues such as bullying and violence (International Council of Nurses, 2021). One significant precursor to these behaviours is incivility, which refers to the breakdown of workplace relations among peers and is distinct from violence, whether physical or verbal, due to its ambiguous intent to harm the victim (Bambi et al., 2018). Incivility not only poisons workplace environments but also diminishes motivation and job satisfaction, ultimately leading to decreased performance and negative repercussions for the nursing profession, patients, organisations and institutional costs (Kline & Lewis, 2019; Kroning, 2019). Workplace violence manifests when there is a discernible intent behind negative behaviours aimed at causing harm. It predominantly commences with psychological harassment, evolving into hostility, encompassing verbal abuse, threats, humiliation, intimidation, criticism, insinuations, social and professional exclusion, discouragement, disinterest and restricted access to information. Instances of workplace violence are observed across hierarchical levels, occurring between senior and junior nurses, as well as among individuals of varying genders. Notably, nurses working in daily shifts are disproportionately affected (Bambi et al., 2018; Bambi, Guazzini et al., 2019; Hartin et al., 2020). Generational differences may exacerbate pre-existing tendencies by experienced nurses to challenge novice nurses (Germann & Moore, 2019; Shorey & Wong, 2021).

Conversely, bullying is characterised by prolonged and abusive behaviour, exacerbated by an intimidating and malicious abuse of power (Anusiewicz et al., 2020; Shorey & Wong, 2021). Lateral violence may occur sporadically, whereas bullying entails a repeated pattern of negative actions, typically recurring weekly or more frequently, persisting for 6 months or longer (Bambi et al., 2018; Bambi, Guazzini et al., 2019; Bambi, Lucchini et al., 2019; Hartin et al., 2019). Among the underlying risk factors are discussions concerning tasks and disagreements regarding nursing care strategies, as well as evaluations of colleagues’ work performance. Potential conflicts and instances of aggression may arise from non-compliance with protocols, inaccurate patient allocation, resource constraints, high nursing workload, a stressful work environment and inadequate leadership (Bambi et al., 2018; Karatuna et al., 2020; Shorey & Wong, 2021).

Victims endure physical, emotional and psychological harm, indirectly compromising patient safety and impacting the quality of care (Karatuna et al., 2020; Shorey & Wong, 2021). The symptoms manifested by individuals who have experienced bullying encompass anxiety, insomnia, asthenia, depression, diminished self-esteem, heightened need for control over daily activities, obsessive preoccupation with work, perception of work-life balance negatively impacting personal life, symptoms indicative of post-traumatic stress disorder, contemplation of leaving the profession and thoughts of resigning (Bambi et al., 2018; Bambi, Guazzini et al., 2019; Karatuna et al., 2020). People frequently hesitate to formally report incidents (Kroning, 2019) due to the perception that organisations do not effectively address them (Hartin et al., 2020). Additionally, nursing leaders are also exposed to bullying, which positively and/or negatively influences their performance (Hampton et al., 2019; Parchment & Andrews, 2019; Parsons et al., 2022; Tuna & Kahraman, 2019).

These phenomena also impact patients. Indeed, as the prevalence of LV among nurses increases, patient safety decreases (Doo & Choi, 2021; Woo & Kim, 2020). This is evidenced by instances where victimised nurses may engage in actions that could potentially compromise patient care and safety (Anusiewicz et al., 2020; Doo & Choi, 2021).

Given the impact on victims and the quality of care, prevention emerges as a paramount concern. Therefore, raising awareness is imperative to stimulate proactive measures aimed at curtailing workplace violence and preventing its escalation into bullying (Bambi et al., 2018; Bambi, Guazzini et al., 2019; Lewis-Pierre et al., 2019). The role of nurse leaders is highlighted as crucial (Shorey & Wong, 2021) because they must work together to create attractive working environments and retain staff by developing and implementing a positive nursing and organisational culture. It was highlighted that unit-based nursing leaders may be aware of bullying and lateral violence but be unsure of the best approaches to address it (Vessey & Williams, 2021). Furthermore, a recent review highlighted that the common goal of the interventions to prevent and deal with bullying in healthcare contexts was to increase the awareness and recognition of bullying among nurses and to develop the ability to respond assertively to uncivil behaviour (Luca et al., 2024). Considering that leaders often find it difficult to identify and deal with bullying, it is crucial for them to acknowledge its presence and be willing to confront it in order to effectively manage it (Hartin et al., 2020). It is important that this happens both in contexts where these phenomena have been poorly explored or measured (Zhang et al., 2022) and in contexts where their presence has been defined, including Swiss healthcare settings (De Barros Lima et al., 2021; Tong et al., 2017).

## Methods

### Aim

To explore the level of knowledge and awareness of nurse leaders of the phenomena of bullying and lateral violence in a public hospital in Switzerland.

### Design

A generic qualitative research approach has been used because it explores and explains facts and phenomena shaped by different perspectives, experiences and organisational, social and cultural contexts (Long & Jiang, 2023).

### Setting and Participants

We used a purposive sampling (Polit & Beck, 2017). The enrolled participants were people working in a Swiss hospital as nurse leaders at different levels and managerial contexts. Participants were eligible if they held a leadership role and were willing to participate in the study.

Participants were recruited on a voluntary basis, and demographic data were collected for each participant. Interviews took place in the workplace, ensuring a comfortable and quiet environment that respected participants’ privacy.

### Study Procedure

The principal investigator acted as the gatekeeper, providing information about the study and consent procedures. Participants were initially contacted via email to determine their interest in participation, followed by a phone call to schedule the interview or focus group appointment. Upon receiving verbal and written information, each participant signed a written informed consent form.

### Data Collection

Data were collected from February to August 2020 through semi-structured interviews and focus groups. To build the grid to guide both of these, two researchers consulted the literature on the topic and validated the tool within the research team. The interviews and focus groups were audio-recorded and facilitated by two qualitative research experts, who alternated between the roles of moderator and observer/writer of field notes. The field notes and memos written in this phase helped ensure reflexivity (Phillippi & Lauderdale, 2018).

### Ethical Considerations

Since this study did not involve patients, it was not necessary to submit it to the Ethical Committee under Swiss legislation (The Federal Council, 2011), but received authorisation from the Faculty Committee (CMSCI_20.01.2020_001). The necessary ethical procedures for research involving human participants were adhered to. Participants were provided with comprehensive information about the project, including its objectives, methods, expected results and associated benefits and risks. Written informed consent was obtained from all participants prior to their involvement. Additionally, measures were implemented to safeguard confidentiality and promote the anonymity of participants.

### Data Analysis

After transcribing the interviews and focus groups verbatim and ensuring participant anonymity by assigning numerical codes, field notes and memos were integrated. Subsequently, two researchers independently conducted a thematic analysis to construct a detailed narrative of the emerging themes from the data. In this respect, the six steps outlined by Braun and Clarke (2006; 2021) were followed.

An external validation phase was carried out by a researcher with expertise in the qualitative method and the phenomena under study. This process resulted in agreement on the identified themes and major themes. Quotes from the participants were included to enhance the reliability of the data. Additionally, to ensure confirmability, a logbook was maintained to document the decision-making process. While the inclusion of detailed results supported transferability, the specificity of the setting posed some challenges. Data saturation was achieved after the fourth focus group and sixth interview.

### Rigour

To ensure the validity and significance of the results, the authors adopted the following strategies:

- Triangulation of methods was employed to confirm emerging conceptualisations and assess phenomena for consistency and constancy;
- Researchers engaged in triangulation and peer debriefing with an external and experienced researcher to review and explore the various aspects of the enquiry in depth.

Considering that guaranteeing rigour and quality in qualitative studies needs an accurate assessment of the qualitative design, sampling details and the process of data collection and analysis, we decided to use the Consolidated Criteria for Reporting Qualitative Studies (Tong et al., 2007). This 32-item checklist is an excellent tool to support transparency in qualitative research.

Finally, the findings can be considered relevant in other contexts due to several factors, including the large sample size, achievement of data saturation and alignment of results with international literature.

## Results

Six interviews (30–50 minutes) and four focus groups (2 h – 2 h and 12 minutes) were conducted. The 35 participants (24 women and 11 men) were between 29 and 59 years old and had an average of 9 years of experience in the role of nurse leaders (Table 1).

**Table 1.** Demographic Data of Participants (*N* = 35).

	Mean (SD), *n* (%)
Age	44.71 ± 7.67
Gender	
Female	24 (69)
Male	11 (31)
Professional role	
Head nurse	30 (86)
Head of department	5 (14)
Working experience as nurse leaders (years)	9.77 ± 8.72
Diploma in advanced studies in healthcare management	
Yes	24 (69)
No	11 (31)

The thematic analysis of the interviews and focus groups revealed 15 themes grouped into five major themes: characteristics of the phenomena, facilitating and hindering factors, emotions and experiences, strategies and supports.

### Characteristics of the Phenomena

The participants described bullying as a negative interaction between people with the aim of putting a person in the position of not being able to express their point of view because of a power imbalance between nurses. The phenomenon could occur among peers, with a superior or a physician and at all levels, but the higher a person was in a hierarchy, the less likely they were to be bullied.

In defining bullying, participants refer to actions such as ‘speaking badly’, often in the absence of the person concerned, looking for a ‘scapegoat’ and creating a truth that did not correspond to the facts. They also noted a tendency to keep leaders uninformed about shared actions through alternative channels (e.g., chats). Leaders questioned how they could have failed to notice the phenomenon, while others, upon becoming aware and identifying it, managed to evade consequences.

The participants linked lateral violence to a person who succeeds in creating consensus in other people but also to denigrating, despising or blaming a person for their errors. This includes speaking negatively about their actions with the intention of undermining the individual and creating a hostile environment. Actions related to resistance to change, divergent views on objectives, and/or unconstructive professional criticism were identified. Additionally, participants highlighted the team's challenges in communicating transparently, politely and without judgement.

Some leaders observed that lateral violence occurred cyclically, and the recurrence of these situations over time led to tolerance, gradually normalising the behaviour of the individual perpetrating the violence. They reported difficulties in recognising these situations, because they often did not occur in their presence, and by the time they became ‘visible’, the situations had already escalated. Those who had directly observed the unfolding of such situations expressed the necessity to comprehend the underlying reasons behind lateral violence behaviours.

Participants identified potential consequences of lateral violence, including isolation, frustration, discomfort, anxiety, stress and depression. They also noted an increase in the affected individual's need for control over their work and a heightened fear of interactions with the colleague perpetrating the violence, as well as apprehension about coming to work. They also discussed consequences in the personal sphere and even for the victim's family. Among the consequences identified were the impact on patient care outcomes, the work environment, the department's reputation and the nursing profession as a whole. Table 2 reports themes and meaningful quotes from participants.

**Table 2.** Major Theme: Characteristics of the Phenomena. Themes and Meaningful Quotes.

Themes	
Bullying	‘The relationship between several people where you cannot express your point of view (…) can lead to complications and losing focus on the objective for which you are working together’. (L4) ‘Generally, it can happen between peers, a boss and his employee, a doctor and a nurse; I think it can occur at all levels’. (L4) ‘I have the impression, linked to the particular situation we are experiencing in the ward, that there is a need to find a group to vent, to find a scapegoat’. (L17) ‘This thing (action against the leader) we would not have known if we had not seen some chats where we were not inside (…) deliberately we were kept in the dark’. (L18) ‘How could I have had such dark eyes?’ (L33) ‘It's something I experienced for several months. Luckily, I didn't have physical problems. I was aware of what was happening’. (L17)
Lateral violence	‘He succeeded in creating consensus (…) the perpetrator had created a group of followers who played deviously and maliciously against the victim, a situation known to everyone’. (L5) ‘Someone (…) saying: “Here it has always been done like that”, leaving no room for discussion’. (L12) ‘I realise that the group struggles to speak directly to each other in a polite and correct manner without making judgements (…)” (L13) ‘In the long term, they thought, “It's the same old colleague”; (…) it was accepted’. (L2) ‘At certain times, he/she is taken as a scapegoat for all situations, and this is cyclical; there are people who are always in the prizes’. (L13) ‘Sometimes they are not visible. The situation has probably already precipitated; (…) it's late’. (L18) ‘I would have liked clarification on this, a chance to confront the person who made this gesture, to understand the root’. (L4)
Potential consequences	‘You create a person who is afraid of everything he does, or doesn't come to work anymore; he goes on sick leave because he survives better at home, at work he throws himself into the pit’. (L5) ‘If the victim suffers all this at work when she goes home, she will not be a happy person (…) the family who has to take her in will also suffer’. (L8) ‘Even the image of the ward, of the service, as far as the hospital (…) is not a good thing for the image you give of your profession’. (L9) ‘One patient [said] “I don't let that one touch me”. I think that's the most serious thing a team can generate: transmitting insecurity to the patient’. (L16)

### Facilitating and Hindering Factors

Facilitating factors of lateral violence/bullying included small teams with low turnover, teams composed of specialised employees and the introduction of a novice within a team. Participants reported also the stability of a group, which led to the establishment of unhealthy relationships among members, a culture of silence surrounding poor behaviour and a lack of moral courage to report misconduct. The hindering factors related to team composition included the knowledge among team members, creation of sensitivity to dialogue and understanding of the phenomenon.

Concerning the organisation, aspects of tolerance towards the phenomenon emerged. These included work stress, heavy workload, perceptions of inequality and judgement and fear in response to novelty.

From a leadership perspective, participants consider actions taken by leaders as facilitating factors: unequal treatment, failure to intervene in a colleague's behaviour, denying the presence of certain behaviours and promoting a hierarchical structure. The absence of leadership recognition was also noted. Additionally, aspects related to the leader's personality, individual stress management approaches, and/or immaturity in handling character incompatibility were identified.

Hindering factors related to leadership included the leader's presence and availability, identification of the weakest employee, sensitivity in dealing with novices and students, consideration of emotions and setting an example for the team. We reported the themes and quotes in Table 3.

**Table 3.** Major Theme: Facilitating and Hindering Factors. Themes and Meaningful Quotes.

Themes	
Team composition	‘Specialised teams without rotation, closed place(s), small team(s); small groups (…)’. (L14) ‘A colleague who cared for critical patients always said: “I'll take care of this one because at least I'm able” ’. (…)” (L12) ‘The exchange in a group must exist. It avoids creating unhealthy relationships in the long term’. (L30) ‘(A) conspiracy of silence (…) fortunately sometimes moral courage comes out’. (L31) ‘When I worked in the ward, when I faced a problem with a colleague, I always thought that before speaking to the head nurse, I would solve it with the colleague and talk to the head nurse when mediation [was] needed’. (L8)
Organisation-related factors	“We have never questioned our culture (…); it is definitely a predominant factor’. (L18) “(…) therefore the unresolved remains there, and day after day, it accumulates and increases’. (L4)
Leadership	‘There are people who can allow any attitude – “that's just the way she is”. (…) This is unacceptable there it is the fault of the head nurse, who should break this vicious circle’. (L10) ‘If you don't want to see them (bullying actions), you put them aside’. (L35) ‘What can favour [bullying] is not recognising the leader or leadership within the ward. If I do not recognise my superior as such, I allow myself to do/say things (…)’ (L20) ‘People are different from each other, and everyone has their way of expressing themselves at the most emotionally charged moments (…)’ (L4) ‘We are the first ones who have to set a good example’. (L18) ‘[Keeping] the discussion open, (…) being present means not underestimating situations (…) being attentive, having regular conversations, (…) being there’. (L17)

### Emotions and Experiences

The experiences of those who had encountered bullying or lateral violence included anger, disappointment, distrust, frustration, stress, exasperation, fear, suffering, feelings of inferiority and worry. Additionally, they sometimes experienced what one participant termed ‘emotional annihilation’. The emotions expressed by leaders who had identified experiences of lateral violence in their teams included annoyance, anger, regret, feelings of failure and fear in the face of the ‘veiled character’ of the phenomenon. They also expressed frustration at not being able to identify those who suffered in silence and astonishment at the extent of lateral violence and bullying within health organisations.

Some participants discussed their realisation of how little these phenomena are visible, while others expressed a reluctance to acknowledge them. The use of social media often renders situations invisible to the head nurse.

Other participants reflected on specific situations, analysing the potential development of relational dynamics. The significance of group management and internal dynamics within groups was also highlighted. Table 4 includes the themes and participants’ meaningful quotes.

**Table 4.** Major Theme: Emotions and Experiences. Themes and Meaningful Quotes.

Themes	
Emotions and experiences of bullied people	‘On an emotional level, it destroys. In a situation like this, it seems that even your skills and your strengths are crumbling. You are no longer the same person; you no longer have the lucidity to say: ‘now I'm going to stop and get things back’ (…) it's a bit like emotional annihilation (…) all the pieces of relationships, the ability to express myself, the demonstration of what I can do fall away’ (L4)
Leaders’ experiences	‘The fact that it is invisible or at least very veiled scares you because you don't realise (…)’ (L20) ‘Some (nurses) interiorise. They are the ones who scare me the most (…) they can't communicate their discomfort’. (L6)
Awareness	‘The definition struck me, the fact that health hierarchies are involved, health contexts. (…) I didn't think it was contextualised to a greater extent in the health field’. (L4) ‘If there's a victim, you don't realise it because I think it's such subtle things that happen for a period, (…) you don't perceive it right away, it's so subtle. (L21) ‘(Bullying) but it's blurry or we don't want to see it?’ (L33) ‘At the beginning it is easy to fall into it. At the moment, you don't think about it, (…) you come up with comments that come out, then the fact that they are fed by the colleague, by the team, it actually results in a huge thing (L8) ‘It's the group dynamics that eventually undergo certain pressures (…) there are also within the debriefings, on the briefings in the handover passages, belittling what the colleague says, trivialising, the little joke out of place’ (L35) ‘I imagine all the chat groups, feeding and fomenting something that will remain absolutely invisible to us, head nurses, but will go on until a situation erupts, and it's late’. (L17)

### Strategies

The initial group of strategies identified from the data analysis focused on recognising possible warning signs, including inadequate reactions and responses to patients and colleagues, nurses isolating themselves, lack of cooperation, inattention, shift changes, requests for help from victims, sudden dismissals and requests for transfers. The second group concerned phenomena observed on a day-to-day basis and included the exclusion of individuals from group activities, discussing someone when they are not present and fairness in the workload distribution within the team.

The prevention of these phenomena is considered fundamental through strategies such as identifying and taking charge of the situation, clearly defining behavioural expectations, setting limits of acceptance, having nursing leadership and sharing personal and institutional values. It is essential to build a network of protection and sensitivity and encourage members to intervene in unhealthy group dynamics. Effective prevention requires leaders to be aware of employees’ characteristics, observe group dynamics, pay attention to employees most at risk and identify warning signs. A significant challenge lies in the leader's intervention focusing on behaviour rather than the individual. The moments identified for implementing these actions include the annual employee evaluation, departmental meetings and debriefings.

The leaders reported taking action by conducting individual interviews or involving all parties, supporting and encouraging victims to speak up, promoting moments of confrontation and awareness within the team and helping those involved understand the consequences of their actions. Table 5 reports themes and meaningful quotes from participants.

**Table 5.** Major Theme: Strategies. Themes and Meaningful Quotes.

Themes	
Warning bells	‘Inadequate reactions, aggressiveness rather than sudden closure, observing how he is in a group [or] how he is with others (…). You breathe a certain air, so you observe a lot’. (L18) ‘You change shifts in order not to work with certain people (…) also the way you talk or approach the ward with both patients and colleagues’. (L25) ‘’When I start hearing a person's name often, even when she is not there, a mechanism is probably being set up that needs to be better observed or analysed’. (L5)
Strategies to prevent the phenomena	“I want to try it, understand it, get my hands on it and do something about it’. (L12) ‘It's more important to do prevention; when the problem is there, it's too late. The head nurse can do a lot in stating the behavioural expectations (…), to what extent they are accepted is a job he can do on a day-to-day basis, in department meetings, in small groups, use several moments, tie in with some event, deepen the discussion of that case, and see how they feel about it’. (L35) ‘If you periodically talk about it, people are more sensitive, and you create that network of protection or reporting’ (L2) ‘Picking up the bells, having this sensitivity (…) I didn't notice, where was I? Someone else noticed a detail I didn't pick up on. As such, it's right that they should point it out to me. It takes training and teamwork. It's a training in seeing, picking up, dealing with these subtle things’. (L34) ‘Develop the annual evaluation as a privileged moment for the employee and explore things you failed to grasp during the year’. (L9) ‘The debriefing moment that we do is critical. You have the opportunity to apologise to the other person, to clarify or to unravel what that moment of tension was, of an over-the-top response’. (L4)
Strategies to solve the situation	‘It is important that the offending party hears what he has injured; only then can he become aware of what his actions cause’. (L17) ‘Interviews must be individual (…) perhaps the victim does not have the moral courage to expose his discomfort in front of the group’. (L15)

### Supports

Participants who had to navigate complex situations appreciated the sensitivity, support and opportunity to share their experiences with the nursing manager. The support from the institution was identified as crucial. Leaders indicated that early recognition of the phenomena and understanding legal and professional aspects are essential skills. Themes and quotes are reported in Table 6.

**Table 6.** Major Theme: Supports. Themes and Meaningful Quotes.

Themes	
The role of leadership	‘The responsibility is shared; it is important to work here: prevention, environment, communication, willingness, maturity in individuals to bring up things that are wrong’. (L31)
Support from the institution	‘Many figures have come out, such as the debriefer and the counsellor (…) the ward manager could have support from these figures’. (L30)
Leaders’ needs	‘It's something you have to fight against that has no form, it has no image, it has no substance. It is difficult to know how many people are experiencing this situation’. (L10) ‘Theoretical training. Starting from a case, do an analysis not only from a management point of view, know what we can do, from various legal, professional and proper behavioural points of view’. (L11)

## Discussion

The primary objective of this research was to evaluate the knowledge nurse leaders possessed regarding the phenomena of bullying and lateral violence. The lack of clarity regarding the terminology surrounding these phenomena is well known in the literature, highlighting the use of interchangeable terms (Bambi, Guazzini et al., 2019). The results clearly revealed the participants’ challenge in defining and distinguishing between the two phenomena. In fact, during interviews and focus groups, nurse leaders reflected on situations and actions experienced or observed, questioning whether they could be attributed to bullying or lateral violence. These phenomena are often described as negative interactions between two or more individuals, with the aim of denigrating, belittling and psychologically harming a person. They involve intentional actions taken to cause harm through negative behaviours (Bambi et al., 2018).

Concerning the temporal nature of bullying, some participants pointed out that dysfunctional behaviour tends to manifest cyclically. Concurrently, existing literature indicates that negative acts of bullying are repeated weekly or more often, over six or more months (Bambi et al., 2018; Bambi, Guazzini et al., 2019; Bambi, Lucchini et al., 2019; Hartin et al., 2019). The participants believed the phenomena could occur at all levels, between professionals, both vertically, between superiors and employees and between peers. However, they did not perceive bullying directed towards nurse leaders. Nevertheless, existing literature does not substantiate this hypothesis. Indeed, Tuna and Kahraman (2019) have highlighted that nurse leaders are also susceptible to bullying, with prevalence rates ranging from 35% to 60%. Such actions can come from their superiors, peers or nurses (Hampton et al., 2019; Parchment & Andrews, 2019).

The participants did not classify behaviours such as ‘bad-mouthing’ and ‘trivialising jokes’ as instances of bullying or lateral violence, risking oversight of the issue and consequently refraining from intervention. Such leadership behaviour aligns with laissez-faire leadership, characterised by leaving space for conflict that fuels the bullying issue (Shorey & Wong, 2021). Moreover, tolerating uncivil behaviour constitutes an ingrained aspect of the prevailing cultural context. This observation corroborates the findings regarding the unawareness of individuals engaging in bullying or lateral violence and the inclination of leaders to deny reality. Nurse leaders highlighted the challenge in noticing such phenomena, as they frequently occur in their absence, and by the time they become apparent, the situation has typically escalated. Indeed, failure to identify uncivil behaviour can initiate spirals of incivility that may escalate into bullying (Bambi, Lucchini et al., 2019). The participants noted that the challenge of observing these phenomena has heightened with the advent of chat rooms and social networks, which can foster dysfunctional situations that go unnoticed by nurse leaders, often resulting in their exclusion. The emergence of new tools for perpetrating violence underscores the professional importance of mutual respect within the workplace. Indeed, the Nurses’ Code of Ethics strongly upholds this value, stating: ‘Nurses respect the privacy and confidentiality of colleagues and those in need of care, and uphold the integrity of the nursing profession in person and all media, including social media’ (International Council of Nurses, 2021).

The findings highlighted that resistance to change poses a significant challenge, particularly within a tightly-knit team. When a novice nurse proposes a change, they often become the primary target of negative behaviours, particularly from experienced nurses. The phenomenon of experienced nurses subjecting novice nurses to ‘testing’ is widely acknowledged, as evidenced by the well-known phrase ‘nurses eat their young’ (André, 2018; Bambi et al., 2018; Hartin et al., 2020; Hawkins et al., 2023). The reverse phenomenon, wherein acts of lateral violence are perpetrated by novices against experienced nurses (Germann & Moore, 2019), was not found within the context under consideration.

Workplace incivility, bullying and lateral violence exert a significant impact on the emotional and psychological well-being of the individuals affected (Bambi et al., 2018; Lewis-Pierre et al., 2019). Participants who reported experiencing bullying or lateral violence during their professional lives expressed a range of emotions, including anger, disappointment, distrust, frustration, stress, exasperation, fear, suffering, a sense of inferiority and worry. The most severe response conveyed was the feeling of ‘emotional annihilation’ experienced by a leader who had been bullied. Recognising the emotional impact is crucial for nurse leaders, as assertively addressing bullying and providing support to those affected can cultivate resilience. This, in turn, enables affected individuals to become ‘wounded healers,’ actively working to dismantle violence in the workplace by fostering strong therapeutic relationships among colleagues (Christie & Jones, 2014; Hampton, 2020).

The fear experienced by participants when confronted with the covert nature of these phenomena was emphasised. This underscores the challenge of leading a team, given the dual role of the head nurse, who serves as the bridge between the institution and the employees. This privileged position entails a continuum of interactions and relationships, necessitating, on one hand, the communication of the institutional vision to the employees and, on the other hand, the establishment of relationships of trust with the employees. Nurse leaders regarded the management role as fundamentally important in preventing these phenomena. They emphasised the sense of ‘co-responsibility’ between the nurse director and nursing middle management in addressing such situations. Nurse leaders started to introspect the dynamics experienced in their wards and analyse the occurrence of dysfunctional behaviours. This prompts the question of whether ‘they are blurred, or we do not want to see them’, or if they are simply perceived as intrinsic to the cultural context. In this regard, Hartin et al. (2020) have emphasised that using culture as an excuse to justify poor behaviour is unacceptable. Participants have also pointed out that inexperienced nursing leaders may lack the necessary tools to identify these phenomena, and it is essential to acknowledge this deficit. However, by identifying the symptoms of a struggling system, nurse leaders should identify and manage bullying behaviours and work with stakeholders to find and implement strategies to mitigate negative behaviour and create respectful workplace behaviours (Hampton et al., 2019; Hawkins et al., 2023).

Awareness prompts leaders to reflect on the maturity of teams based on values, the challenge of integrating novice nurses, and change, which should not be perceived as a threat but as a ‘nurturing evolution’. The process of raising awareness leads leaders to consider the significance of their role in addressing dysfunctional team dynamics. It becomes essential for nurse leaders to maintain a high level of attention because failure to do so could result in victims of bullying being reluctant to report their bullies and may foster feelings of distrust towards the leader, thereby perpetuating an ineffective management cycle of the phenomenon and its normalisation. Consequently, this can lead to victims leaving the workplace (Galanis et al., 2024) due to a sense of betrayal by the organisation (Brewer et al., 2020; Hartin et al., 2020; Kroning, 2019).

The participants in this study noted that bullying and lateral violence could also have implications for the victim's personal life and family. Moreover, the reputation of the ward and the nursing profession can be tarnished; the phenomenon is seen as an epidemic that adversely affects the entire team. The same connotation is expressed in the literature, acknowledging the ‘epidemic’ nature of these phenomena among nursing staff and their detrimental impact on the workplace environment (Germann & Moore, 2019; Kroning, 2019). The significant effect of these phenomena is evident in both the well-being of employees and the financial sustainability of a hospital. This underscores the urgency with which nurse leaders must address and prevent them, as well as advocate for research into their financial costs within their institutions (Bambi et al., 2018; Bambi, Guazzini et al., 2019; Lever et al., 2019; Lewis-Pierre et al., 2019). Many leaders believe that such phenomena influence care outcomes and patients, prompting reflection on the potential impact they might also have on patient safety and the quality of care within the hospital (Doo & Choi, 2021; Kroning, 2019; Woo & Kim, 2020).

Nurse leaders deem it essential to articulate their expectations to cultivate a cohesive team culture clearly. Within such a culture, nurses are empowered to be the first to address uncivil behaviour, demonstrating moral courage, expertise, humility, respect, consideration and attentiveness. Indeed, nurses are responsible for demonstrating care and respect for one another and integrating acts of kindness towards others into their daily routines (Hawkins et al., 2023). The role of nurse leaders is crucial in encouraging staff members to report observed uncivil behaviour proactively and to implement strategies to mitigate its consequences (Kim, 2020; MacCurtain et al., 2018). In this way, prevention transforms into a collective effort, fostering a culture of openness among employees. Indeed, eradicating these phenomena in healthcare settings necessitates the active involvement of nurses at all levels, alongside support from nurse leaders, organisations and health policies (Luca et al., 2024). A zero-tolerance culture, where employee feedback and reporting are highly valued and acted upon, is crucial. By modelling good behaviour and communication, individuals are held accountable for any acts of incivility (Kroning, 2019; Millis, 2020).

## Strengths and Limitations

This qualitative research was conducted within a specific context. Considering the setting and location of the study, the results may not be broadly generalisable. However, with careful consideration of various contextual nuances, they can be transferable to other settings. It is important to note that while the results are interesting, replicating them in culturally and organisationally diverse contexts may pose challenges due to the contextual nature of bullying and lateral violence. Nonetheless, these limitations do not diminish the richness of the data and findings, as they shed light on the awareness of bullying within a public Swiss hospital for the first time.

## Implications for Practice

The findings of this study can provide valuable insights for middle management in understanding the significance of recognising bullying and lateral violence, along with the factors that contribute to their emergence from the early stages. Understanding how nurses perceive these phenomena is crucial for nursing leadership and clinical nursing managers. They can utilise the information from this study to support nurses and cultivate conditions that enhance their working environment and interpersonal relations in clinical practice. For instance, they could arrange periodic meetings to gather and discuss emerging situations, as well as borderline cases, aiming to raise awareness among all professionals within the work teams regarding these issues and associated risks. Collaboratively, they can identify potential organisational-level solutions.

## Conclusion

As bullying and lateral violence increasingly permeate healthcare organisations, with the nursing profession being particularly susceptible, this study aimed to assess the awareness and knowledge levels of these phenomena among nursing managers in a Swiss public hospital. The findings reveal that nursing managers may lack awareness of incivility, bullying and lateral violence, struggling to identify them effectively. This lack of recognition often stems from an inability to adopt a systemic approach to these complex issues. The awareness gained by head nurses during data collection underscored their need for knowledge, as highlighted by the statement ‘it would be interesting to know what you are fighting against’.

The research results highlighted the importance of considering the impact of these phenomena on employees’ well-being, their implications in both professional and personal spheres, and the potential consequences on patient safety, quality of care and financial performance. The study demonstrates the necessity of promoting a preventive approach by enhancing nursing managers’ competency in observing everyday work realities and identifying strategies to address bullying.

However, addressing this challenge is not straightforward. Providing care with humanity and empathy within an increasingly complex system, characterised by limited resources, conflicts, aggression, defensive medicine, technological advancements and ethical dilemmas, poses significant difficulties for nurses. In this context, healthcare organisations must shoulder responsibility for preventing bullying and lateral violence. By addressing these phenomena, organisations can cultivate a work environment where nurses feel valued, motivated and rewarded. Nurse leaders bear a moral duty to promote nurses’ well-being, which can positively impact the quality and safety of care and patient well-being, and an institutional and societal duty, considering the costs associated with work-related stress.

Simultaneously, fostering a zero-tolerance environment for uncivilised behaviour is crucial. Moreover, promoting new research on developing and implementing specific interventions to prevent and address these phenomena is imperative.

## References

[bibr1-23779608241274210] André S. (2018). Embracing generational diversity: Reducing and managing workplace conflict. ORNAC Journal, 36(4), 13–35. https://search.ebscohost.com/login.aspx?direct=true&db=c8h&AN=133410687&site=ehost-live

[bibr2-23779608241274210] Anusiewicz C. V. Ivankova N. V. Swiger P. A. Gillespie G. L. Li P. Patrician P. A. (2020). How does workplace bullying influence nurses’ abilities to provide patient care? A nurse perspective. Journal of Clinical Nursing, 29(21-22), 4148–4160. 10.1111/jocn.15443 32757394 PMC8040339

[bibr3-23779608241274210] Bambi S. Foa C. De Felippis C. Lucchini A. Guazzini A. Rasero L. (2018). Workplace incivility, lateral violence and bullying among nurses. A review about their prevalence and related factors. Acta Bio-Medica, 89(6-S), 51–79. 10.23750/abm.v89i6-S.7461 30038204 PMC6357596

[bibr4-23779608241274210] Bambi S. Guazzini A. De Felippis C. Lucchini A. Rasero L. (2017, November 30). Preventing workplace incivility, lateral violence and bullying between nurses a narrative literature review. Acta Bio-Medica, 88(5s), 39–47. 10.23750/abm.v88i5-S.6838 PMC6357576 29189704

[bibr5-23779608241274210] Bambi S. Guazzini A. Piredda M. Lucchini A. De Marinis M. G. Rasero L. (2019). Negative interactions among nurses: An explorative study on lateral violence and bullying in nursing work settings. Journal of Nursing Management, 27(4), 749–757. 10.1111/jonm.12738 30506602

[bibr6-23779608241274210] Bambi S. Lucchini A. Guazzini A. Caruso C. Rasero L. (2019). Workplace incivility, horizontal violence, bullying and mobbing among peers in nursing. Theories and Interpretation Models. Professioni Infermieristiche, 72(3), CP1–CP8. 10.7429/pi.2019.3cp001

[bibr7-23779608241274210] Braun V. Clarke V. (2006). Using thematic analysis in psychology. Qualitative Research in Psychology, 3(2), 77–101. 10.1191/1478088706qp063oa

[bibr8-23779608241274210] Braun V. Clarke V. (2021). One size fits all? What counts as quality practice in (reflexive) thematic analysis? Qualitative Research in Psychology, 18(3), 328–352. 10.1080/14780887.2020.1769238

[bibr9-23779608241274210] Brewer K. C. Oh K. M. Kitsantas P. Zhao X. (2020, January). Workplace bullying among nurses and organizational response: An online cross-sectional study. Journal of Nursing Management, 28(1), 148–156. 10.1111/jonm.12908 31746065

[bibr10-23779608241274210] Christie W. Jones S. (2014). Lateral violence in nursing and the theory of the nurse as wounded healer. OJIN: The Online Journal of Issues in Nursing, 19(1), 1. 10.3912/OJIN.Vol19No01PPT01 26812194

[bibr11-23779608241274210] Crawford C. L. Chu F. Judson L. H. Cuenca E. Jadalla A. A. Tze-Polo L. Kawar L. N. Runnels C. Garvida R. (2019). An integrative review of nurse-to-nurse incivility, hostility, and workplace violence: A GPS for nurse leaders. Nursing Administration Quarterly, 43(2), 138–156. 10.1097/NAQ.0000000000000338 30839451

[bibr12-23779608241274210] De Barros Lima C. Davitti V. Goncalves S. Piattini S. Levati S. D'Angelo V. Prandi C. Bianchi M. (2021). Il bullismo nel sistema sanitario ticinese: L’impatto sul benessere dei collaboratori e sull’organizzazione [Bullying in Ticino's healthcare contexts: Impact on employee's well being and organization]. Professioni Infermieristiche, 74(3), 131–138. https://doi-org.eoc.swissconsortium.ch/10.7429/pi.2021.742131 35084155 10.7429/pi.2021.742131

[bibr13-23779608241274210] Doo E. Y. Choi S. (2021). Effects of horizontal violence among nurses on patient safety: Mediation of organisational communication satisfaction and moderated mediation of organisational silence. Journal of Nursing Management, 29(3), 526–534. 10.1111/jonm.13182 33053246

[bibr14-23779608241274210] The Federal Council. (2011). *Federal act on research involving human beings*. Retrieved August 10, 2022, from https://www.fedlex.admin.ch/eli/cc/2013/617/en

[bibr15-23779608241274210] Galanis P. Moisoglou I. Katsiroumpa A. Malliarou M. Vraka I. Gallos P. Kalogeropoulou M. Papathanasiou I. V. (2024). Impact of workplace bullying on quiet quitting in nurses: The mediating effect of coping strategies. Healthcare (Basel, Switzerland), 12(7), 797. https://doi-org.eoc.swissconsortium.ch/10.3390/healthcare12070797 38610219 10.3390/healthcare12070797 PMC11011316

[bibr16-23779608241274210] Germann S. Moore S. (2019). Lateral violence, a nursing epidemic? Reflections on Nursing Leadership, 45(2), 47–51. https://search.ebscohost.com/login.aspx?direct=true&db=c8h&AN=138841174&site=ehost-live

[bibr17-23779608241274210] Hampton D. (2020). The importance of resilience in helping leaders cope with psychological violence/workplace bullying. Kentucky Nurse, 68(1), 10–10. https://search.ebscohost.com/login.aspx?direct=true&db=c8h&AN=141281300&site=ehost-live

[bibr18-23779608241274210] Hampton D. Kay Rayens M. Tharp-Barrie K. (2019). Experience of nursing leaders with workplace bullying and how to best cope. Journal of Nursing Management, 27(3), 517–526. 10.1111/jonm.12706 30136408

[bibr19-23779608241274210] Hartin P. Birks M. Lindsay D. (2019). Bullying in nursing: Is it in the eye of the beholder? Policy, Politics, & Nursing Practice, 20(2), 82–91. 10.1177/1527154419845411 31060477

[bibr20-23779608241274210] Hartin P. Birks M. Lindsay D. (2020). Bullying in nursing: How has it changed over 4 decades? Journal of Nursing Management, 28(7), 1619–1626. 10.1111/jonm.13117 32745349

[bibr21-23779608241274210] Hawkins N. Jeong S. Y. Smith T. Sim J. (2023). A conflicted tribe under pressure: A qualitative study of negative workplace behaviour in nursing. Journal of Advanced Nursing, 79(2), 711–726. 10.1111/jan.15491 36394212 PMC10100446

[bibr22-23779608241274210] International Council of Nurses. (2021). *ICN code of ethics for nurses*. Retrieved March 23, 2022, from https://www.icn.ch/system/files/2021-10/ICN_Code-of-Ethics_EN_Web_0.pdf

[bibr23-23779608241274210] Karatuna I. Jonsson S. Muhonen T. (2020, November). Workplace bullying in the nursing profession: A cross-cultural scoping review. International Journal of Nursing Studies, 111, 103628. 10.1016/j.ijnurstu.2020.103628 32932063

[bibr24-23779608241274210] Kim K. (2020). Exploring the influence of workplace violence and bystander behaviour on patient safety in Korea: A pilot study. Journal of Nursing Management, 28(3), 735–743. 10.1111/jonm.12991 32124510

[bibr25-23779608241274210] Kline R. Lewis D. (2019). The price of fear: Estimating the financial cost of bullying and harassment to the NHS in England. Public Money & Management, 39(3), 166–174. 10.1080/09540962.2018.1535044

[bibr26-23779608241274210] Kroning M. (2019). Be CIVIL: Committing to zero tolerance for workplace incivility. Nursing Management, 50(10), 52–54. 10.1097/01.NUMA.0000580628.91369.50 31567826

[bibr27-23779608241274210] Lever I. Dyball D. Greenberg N. Stevelink S. A. M. (2019). Health consequences of bullying in the healthcare workplace: A systematic review. Journal of Advanced Nursing, 75(12), 3195–3209. 10.1111/jan.13986 30816567

[bibr28-23779608241274210] Lewis-Pierre L. Anglade D. Saber D. Gattamorta K. A. Piehl D. (2019). Evaluating horizontal violence and bullying in the nursing workforce of an oncology academic medical center. Journal of Nursing Management, 27(5), 1005–1010. 10.1111/jonm.12763 30793404

[bibr29-23779608241274210] Long Q. Jiang H. (2023). Qualitative research in health: Value and visibility. The Lancet Regional Health – Western Pacific, 34. 10.1016/j.lanwpc.2023.100790 PMC10240377 37283968

[bibr30-23779608241274210] Luca C. E. Sartorio A. Bonetti L. Bianchi M. (2024). Interventions for preventing and resolving bullying in nursing: A scoping review. Healthcare (Basel, Switzerland), 12(2), 280. 10.3390/healthcare12020280 38275560 PMC10815476

[bibr31-23779608241274210] MacCurtain S. Murphy C. O'Sullivan M. MacMahon J. Turner T. (2018). To stand back or step in? Exploring the responses of employees who observe workplace bullying. Nursing Inquiry, 25(1), e12207. 10.1111/nin.12207 28631389

[bibr32-23779608241274210] Millis S. (2020). Changing workplace culture: What would it take to speak up? Journal of Perioperative Nursing, 33(1), 15–18. 10.26550/2209-1092.1078

[bibr33-23779608241274210] Parchment J. Andrews D. (2019). The incidence of workplace bullying and related environmental factors among nurse managers. The Journal of Nursing Administration, 49(3), 132–137. 10.1097/nna.0000000000000726 30789556

[bibr34-23779608241274210] Parsons K. Gaudine A. Patrick L. Busby L. (2022). Nurse leaders’ experiences of upwards violence in the workplace: A qualitative systematic review. JBI Database of Systematic Reviews and Implementation Reports, 20(5), 1243–1274. 10.11124/jbies-21-00078 34889309

[bibr35-23779608241274210] Phillippi J. Lauderdale J. (2018). A guide to field notes for qualitative research: Context and conversation. Qualitative Health Research, 28(3), 381–388. 10.1177/1049732317697102 29298584

[bibr36-23779608241274210] Polit D. F. Beck C. T. (2017). Nursing research: Generating and assessing evidence for nursing practice. Wolters Kluwer Health. https://books.google.ch/books?id=O7HRoQEACAAJ

[bibr37-23779608241274210] Shorey S. Wong P. Z. E. (2021). A qualitative systematic review on nurses’ experiences of workplace bullying and implications for nursing practice. Journal of Advanced Nursing, 77(11), 4306–4320. 10.1111/jan.14912 34081351

[bibr38-23779608241274210] Tong A. Sainsbury P. Craig J. (2007). Consolidated criteria for reporting qualitative research (COREQ): A 32-item checklist for interviews and focus groups. International Journal for Quality in Health Care: Journal of the International Society for Quality in Health Care, 19(6), 349–357. 10.1093/intqhc/mzm042 17872937

[bibr39-23779608241274210] Tong M. Schwendimann R. Zúñiga F. (2017). Mobbing among care workers in nursing homes: A cross-sectional secondary analysis of the Swiss Nursing Homes Human Resources Project. International Journal of Nursing Studies, 66, 72–81. 10.1016/j.ijnurstu.2016.12.005 28017895

[bibr40-23779608241274210] Tuna R. Kahraman B. (2019). Workplace bullying: A qualitative study on experiences of Turkish nurse managers. Journal of Nursing Management, 27(6), 1159–1166. 10.1111/jonm.12787 31063636

[bibr41-23779608241274210] Vessey J. A. Williams L. (2021). Addressing bullying and lateral violence in the workplace: A quality improvement initiative. Journal of Nursing Care Quality, 36(1), 20–24. 10.1097/NCQ.0000000000000480 32168113

[bibr42-23779608241274210] Woo C. H. Kim C. (2020). Impact of workplace incivility on compassion competence of Korean nurses: Moderating effect of psychological capital. Journal of Nursing Management, 28(3), 682–689. 10.1111/jonm.12982 32072694

[bibr43-23779608241274210] Zhang Y. Cai J. Yin R. Qin S. Wang H. Shi X. Mao L. (2022). Prevalence of lateral violence in nurse workplace: A systematic review and meta-analysis. BMJ Open, 12(3), e054014. 10.1136/bmjopen-2021-054014 PMC8966576 35351708

